# Backbone tuning in indenylidene–ruthenium complexes bearing an unsaturated *N*-heterocyclic carbene

**DOI:** 10.3762/bjoc.6.128

**Published:** 2010-11-23

**Authors:** César A Urbina-Blanco, Xavier Bantreil, Hervé Clavier, Alexandra M Z Slawin, Steven P Nolan

**Affiliations:** 1 EaStCHEM School of Chemistry, University of St Andrews, St Andrews KY16 9ST, United Kingdom; 2 present address: Institut des Sciences Moléculaires de Marseille Université Aix-Marseille, UMR CNRS 6263, France

**Keywords:** *N*-heterocyclic carbene, olefin metathesis, percent buried volume, ruthenium–indenylidene, Tolman electronic parameter

## Abstract

The steric and electronic influence of backbone substitution in IMes-based (IMes = 1,3-bis(2,4,6-trimethylphenyl)imidazol-2-ylidene) *N*-heterocyclic carbenes (NHC) was probed by synthesizing the [RhCl(CO)_2_(NHC)] series of complexes to quantify experimentally the Tolman electronic parameter (electronic) and the percent buried volume (%*V*_bur_, steric) parameters. The corresponding ruthenium–indenylidene complexes were also synthesized and tested in benchmark metathesis transformations to establish possible correlations between reactivity and NHC electronic and steric parameters.

## Introduction

**Figure 1:**
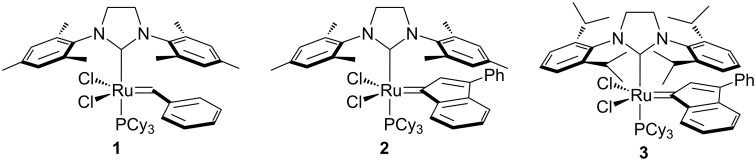
Representative olefin metathesis catalysts.

The use of *N*-heterocyclic carbenes (NHC) as spectator ligands in ruthenium-mediated olefin metathesis represents one of the most important breakthroughs in this field [1–8]. Mixed complexes bearing both a phosphane and a NHC ligand, so-called 2^nd^ generation catalysts, typically display better thermal stability and activities compared to 1^st^ generation catalysts [9–10]. Key to the success and research activity involving 2^nd^ generation catalysts has been the wide selection of NHCs available [11–12]. These highly basic ligands have now been featured in a number of catalysts that display excellent activity in olefin metathesis. NHCs have become the ligand *par excellence* in olefin metathesis (Figure 1) [7–8].

**Figure 2:**
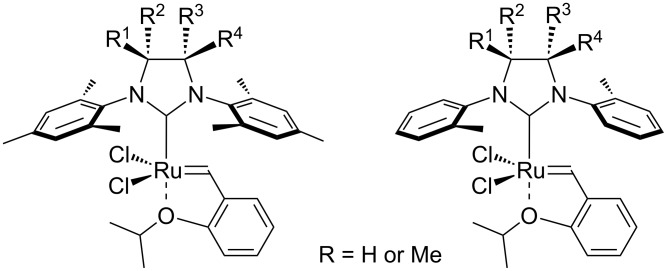
Highly active olefin metathesis catalysts bearing NHC with backbone substitution.

In order to improve catalytic activity, the possibility of fine-tuning of NHC steric and electronic properties has been exploited. Bulkier and more electron-donating NHCs allow faster initiation with usually a concurrent increase in reaction rate when the olefin substrate is of low steric hindrance [13–17]. Less sterically demanding NHCs are typically used for the synthesis of highly encumbered olefins [18]. Recent studies have shown that backbone substitution in saturated NHCs greatly improves catalyst stability by restricting rotation around the *N*–C_aryl_ bond (Figure 2); this presumably slows catalyst decomposition via an observed C–H activation route [19].

These results encouraged us to explore the electronic influence of backbone substitution in unsaturated NHCs with ruthenium–indenylidene complexes. Indenylidene catalysts are rapidly becoming quite popular [20–21], due to the availability of ruthenium precursors [22] and their straightforward synthesis. The higher steric hindrance and improved electronic donor ability of the indenylidene moiety also contribute to the observed increased stability compared to benzylidene congeners. This family of complexes displayed interesting stability even when forcing reaction conditions are required [13,23–25].

Herein, we present the synthesis and characterization of three new ruthenium–indenylidene catalysts and their performance in benchmark metathesis transformations. In order to quantify the Tolman electronic parameter associated with IMes-type (IMes = 1,3-bis(2,4,6-trimethylphenyl)imidazol-2-ylidene) ligands possessing variable backbone substitution patterns, the corresponding series of [RhCl(CO)_2_(NHC)] complexes was synthesized. X-ray diffraction studies permit the determination of the percent buried volume (%*V*_bur_) of these NHCs ligands and quantify their respective steric parameter.

## Results and Discussion

### Evaluation of the ligand electronic and steric properties

Previous studies have shown that the electronic parameter of NHC (and other) ligands can be quantified employing the stretching frequency of CO (ν_CO_) in various transition metal–carbonyl complexes [26–32]. This method was initially developed by Tolman [33], using the average infrared frequency of CO in [Ni(CO)_3_L] complexes. This electronic parameter has become known as the Tolman electronic parameter (TEP) and has been used to quantify the electron donor ability of phosphanes, and more recently, has been used to study the electronic properties of NHCs [34].

However, the high toxicity of [Ni(CO)_4_] encouraged the search for analogous systems using different metals to determine the TEP. One of the most popular and suitable alternatives to nickel is a rhodium carbonyl system, since it is easily synthesised and handled [34]. In this work, a series of [RhCl(CO)_2_(NHC)] complexes were synthesized in order to evaluate the electronic donor ability of the NHCs.

**Scheme 1:**
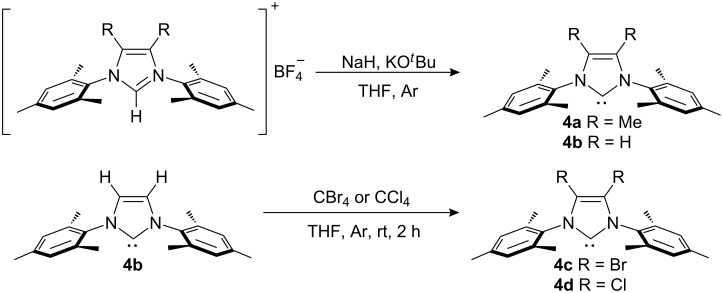
Synthesis of the free NHCs.

The free carbenes were prepared according to literature procedures. Free IMes (**4b**) [35] and IMesMe (**4a**) [36] were synthesized from the corresponding tetrafluoroborate salts; free IMesBr (**4c**) [37] and IMesCl (**4d**) [38] were synthesized in situ prior to complex synthesis by reacting free IMes with CBr_4_ and CCl_4_, respectively (Scheme 1).

**Scheme 2:**
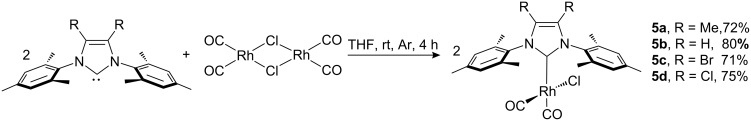
Synthesis of [RhCl(CO)_2_(NHC)] complexes.

The complexes **5a**–**d** were prepared by reacting [Rh(CO)_2_Cl]_2_ with the corresponding free carbene in THF (Scheme 2). After stirring for 4 h at room temperature, removal of the solvents and washing of the residue with pentane, the corresponding complexes were obtained as yellow microcrystalline solids, in good yields (71–80%).

Infrared spectra were recorded in DCM for **5a**–**d** and the carbonyl stretching frequencies (ν_COav_) were used to provide the TEP (Table 1). As expected, the backbone substitution pattern has a profound effect on the electronic donor capacity of the NHC, and a linear correlation between the electronegativity of the backbone substituent (measured as the Hammett parameter, σ_p_) and the average carbonyl stretching frequency (ν_COav_) in [RhCl(CO)_2_(NHC)] complexes is observed (R^2^ = 0.98).

**Table 1:** Electronic and steric parameters of NHCs in [RhCl(CO)_2_(NHC)] complexes.

Complex	ν_COav_ (cm^−1^)	TEP^a^ (cm^−1^)	σ_p_	%*V*_bur_

[RhCl(CO)_2_(IMesMe)]	2034.8	2048.0	−0.170	31.7 ± 0.1^b^
[RhCl(CO)_2_(IMes)]	2037.6	2050.3	0.000	31.8 ± 0.5^b^
[RhCl(CO)_2_(IMesBr)]	2041.3	2053.3	0.227	32.6
[RhCl(CO)_2_(IMesCl)]	2042.5	2054.2	0.232	32.7

^a^TEP calculated using equation TEP = 0.8001 ν_COav_ + 420.0 cm^−1^. ^b^Average of the independent structures.

The electron donating nature of the NHC decreases along the series: IMesMe > IMes > IMesBr > IMesCl. As an internal check of the data, it is worth noting that the calculated TEP for IMes (2050.3 cm^−1^) agrees well with the experimentally obtained value in the nickel system (2051.5 cm^−1^) [34].

Given their steric and geometric variability, evaluating the steric parameters of NHCs poses a more challenging task. One of the more recent methodologies defines a percent buried volume (%*V*_bur_), which quantifies the volume of a sphere centred around the metal (with a specific radius distance) occupied by the ligand. The more sterically demanding ligands will correspond to larger %*V*_bur_ values [36,39].

Analysis of the crystal structures of **5a**–**d**, in conjunction with the aforementioned computational tool, allow us to conclude that a hydrogen–methyl or hydrogen–halogen exchange in the backbone creates small steric variation in the NHC evidenced by the very close values obtained for the %*V*_bur_ [40]. However, the %*V*_bur_ for the ligands correlates very well with the size of the substituent: IMesCl ≈ IMesBr > IMes ≈ IMesMe.

### Synthesis of ruthenium–indenylidene catalysts and their performance in olefin metathesis

**Scheme 3:**
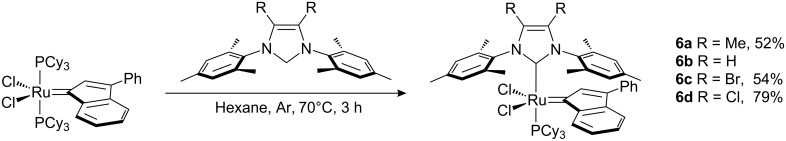
Synthesis of [RuCl_2_(NHC)(PCy_3_)(Ind)] complexes.

The ruthenium–indenylidene complexes were synthesized in order to establish how strongly the electronic and steric parameters of the NHC influence catalytic activity in olefin metathesis. As reported for **6b** [41], precatalysts **6a**, **6c** and **6d** were synthesized by exchange between PCy_3_ and the corresponding free carbene in [RuCl_2_(PCy_3_)_2_(Ind)] (Scheme 3). The new complexes proved challenging to purify by recrystallization, however flash column chromatography on silica gel afforded highly pure compounds in moderate yields (52–79%). The use of this purification technique also attests to the robustness of the novel complexes.

Complexes **6a**, **6c** and **6d** are stable in the solid state under aerobic conditions and exhibit remarkable stability in solution under inert atmosphere. ^1^H NMR analysis of their solutions showed little decomposition even after 24 h in dichloromethane-*d*_2_ at 40 °C. Traces of degradation could be observed after 1 h in toluene at 80 °C with complete decomposition after 24 h.

Complexes **6a**–**d** were then tested in benchmark metathesis transformations with substrates featuring different steric properties (Table 2). The catalysts were found to perform very modestly in the synthesis of poorly hindered substrates **7b** and **8b** at room temperature, but their performance improves significantly upon thermal activation. Thus, **6d** achieves full conversion within 2 h at 80 °C. Similar results were achieved with substrates **9a**–**12a.** Interestingly transformations at room temperature exhibit no correlation between the electronic properties of the carbene and the catalytic outcome. However, more challenging substrates that effect the formation of tetrasubstituted double bonds do present a trend. Even if catalysts performed similarly, the highest conversions were constantly reached with the catalyst bearing the least electron-donating carbene, namely IMesCl (**6d**). These results can be rationalized in terms of the mechanism of the reaction. Although a more electron-donating NHC should better stabilize the 14-electron active species and allow better catalytic activity, the faster initiation is also related to faster catalyst decomposition; at 80 °C, this deactivation contributes considerably to the catalytic outcome. In conclusion, we suggest that **6d** represents the most advantageous catalyst owing to its improved stability, which is attributed to reduced initiation from poorer electron-donating ability of the NHC ligand.

**Table 2:** Catalytic evaluation of **6a–d** in benchmark metathesis transformations.^a^

Substrate	Product	Catalyst	Loading (mol %)	*T* (°C)	Time (h)	Conv^b^ (%) (yield (%))

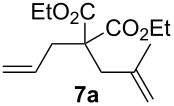	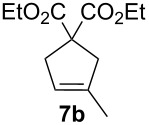	**6a** **6b** **6c** **6d**	1	rt^c^	24	22 49 9 3
		**6d**		80	2	<99 (95)

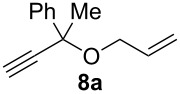	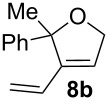	**6a** **6b** **6c** **6d**	1	rt^c^	24	33 39 65 33
		**6d**		80	2	<99 (97)

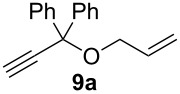	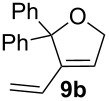	**6d**	1	80	2	<99 (98)

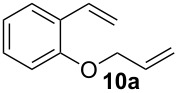	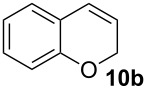	**6d**	1	80	2	<99 (85)

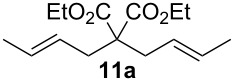	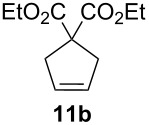	**6d**	1	80	2	<99 (96)

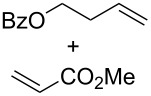	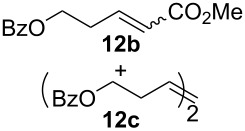	**6d**	1	80	5	**b**: 69 (55) E/Z > 20:1 **c**: 9

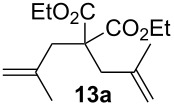	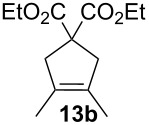	**6a** **6b** **6c** **6d**	5	80	5	62 37 69 78 (72)

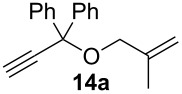	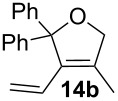	**6a** **6b** **6c** **6d**	5	80	2	31 36 18 43

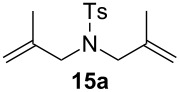	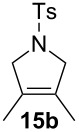	**6a** **6b** **6c** **6d**	2	80	3	58 86 98 98 (95)

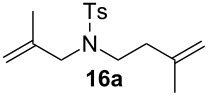	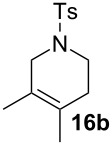	**6a** **6b** **6c** **6d**	2	80	3	90 97 99 99 (99)

^a^Reaction conditions: substrate (0.5 mmol), toluene (0.1 M), N_2_, 80 °C. ^b^Conversions determined by ^1^H NMR. ^c^DCM (0.1 M).

## Conclusion

The effects of modulating the nature of substituents on the backbone (C4 and C5) positions of the IMes ligand have permitted a quantification of the electronic and steric parameters associated with these synthetic variations. Using a rhodium carbonyl system, the electronic variations brought about by substituents on the NHC lead to the following ligand electronic donor scale: IMesMe > IMes > IMesBr > IMesCl. The size of the substituent also affects the steric hindrance of the ligands, and the percent buried volume of the NHCs decreases in the following order: IMesCl ≈ IMesBr > IMes ≈ IMesMe. A modest trend between the electronic properties of the carbene and the catalytic outcome was found in the synthesis of tetrasubstituted olefin. This was attributed to improved stability of the catalyst derived from lower electron-donating properties of the NHC.

## Supporting Information

Detailed experimental procedures for the synthesis of complexes **5a**–**d**, **6a**, **6c** and **6d**, and procedures for the catalysis are available in the supporting information. The CIF files of crystal structures **5a**–**d** have been deposited with the CCDC, No. 793640–793643, respectively. Copies of the data can be obtained free of charge on applications to CCDC, 12 Union Road, Cambridge CB2 1EZ, UK, fax: +44 1223 336 033; http://www.ccdc.cam.ac.uk; e-mail: deposit@ccdc.cam.ac.uk.

File 1 Supporting Information.
