# Supplementary material for: Backbone tuning in indenylidene–ruthenium complexes bearing an unsaturated N-heterocyclic carbene
Source: Beilstein J Org Chem. 2010 Nov 23;6:1120–6. doi: 10.3762/bjoc.6.128 (PMC3002078; doi:10.3762/bjoc.6.128)
Supplement: File 1 — Supporting Information. [file Beilstein_J_Org_Chem-06-1120-s001.pdf]

Supporting Information

for

Backbone tuning in indenylidene–ruthenium complexes bearing an unsaturated *N*-heterocyclic carbene

César A. Urbina-Blanco,[1] Xavier Bantreil,[1] Hervé Clavier,[1,2] Alexandra M. Z. Slawin,[1] and Steven P. Nolan*[1]

Address: [1]EaStCHEM School of Chemistry, University of St Andrews, St Andrews KY16 9ST, United Kingdom and [2]present address: Institut des Sciences Moléculaires de Marseille Université Aix-Marseille, UMR CNRS 6263, France

Email: Steven P. Nolan* - snolan@st-andrews.ac.uk

*Corresponding author

General considerations: All reagents were used as received. Dichloromethane and toluene were dispensed from a solvent purification system from Innovative Technology. Catalyst syntheses were performed in a MBraun glovebox containing dry argon and less than 1 ppm oxygen. ^{1}H, ^{31}P, and ^{13}C NMR spectra were recorded on a Bruker Avance 300 or a Bruker Avance II 400 Ultrashield NMR spectrometers. Elemental analyses were performed by the University of St Andrews Analytical Service. IR measurements were performed in a Perkin Elmer Spectrum GX IR spectrometer. Complex **6b** [1] and substrates and products **7-9** [2], **10** [3]**, 11** [4]**, 12** [5]**, 13** [2]**, 14** [6] **15–16** [2] have been previously described in the literature.

Synthesis of [RhCl(CO)$_2$(IMesMe)] (5a)

In the glovebox, in a vial with a solution of [Rh(CO)$_2$Cl]$_2$ (50 mg, 0.127 mmol) in 5 mL of THF, a solution of free IMesMe (85 mg, 0.255 mmol) was added dropwise, the reaction mixture was stirred for 4 h, taken out of the glovebox and the solvents removed under vacuum. The remaining solid was washed with pentane (3 x 10 mL) and dried under vacuum to afford **4a** as a pale yellow solid (96.3 mg, 0.183 mmol, 72%). Suitable crystals for single X-ray diffraction were grown by vapour diffusion of pentane into a concentrated solution of **4a** in DCM.

^{1}H NMR (CD$_2$Cl$_2$, 300 MHz): δ = 7.05 (s, 4H, H^{Ar}), 2.39 (s, 6H, *p*-CH$_3^{Mes}$), 2.10–2.18 (m, 12H, *o*-CH$_3^{Mes}$), 1.86 ppm (s, 6H). ^{13}C NMR (CD$_2$Cl$_2$, 75 MHz) δ ppm 185.83 (d, *J* = 53.99 Hz, 1C) 183.53 (d, *J* = 74.34 Hz, 1C) 173.33 (d, *J* = 44.60 Hz, 1C) 139.58 (s, 2C) 136.17 (s, 4C) 134.01 (s, 2C) 129.55 (s, 4CH) 127.55 (s, 2C) 21.31 (s, 2CH$_3$) 18.50 (s, 4CH$_3$) 9.22 (s, 5CH$_3$). IR (ν_{CO}) in CH$_2$Cl$_2$: 2076.98, 1992.54 cm^{-1}. Anal. calcd for C$_{25}$H$_{30}$ClN$_2$O$_2$Rh (MW 528.88): C, 56.77; H, 5.72; N, 5.30. Found: C, 56.35; H, 5.27; N, 5.15.

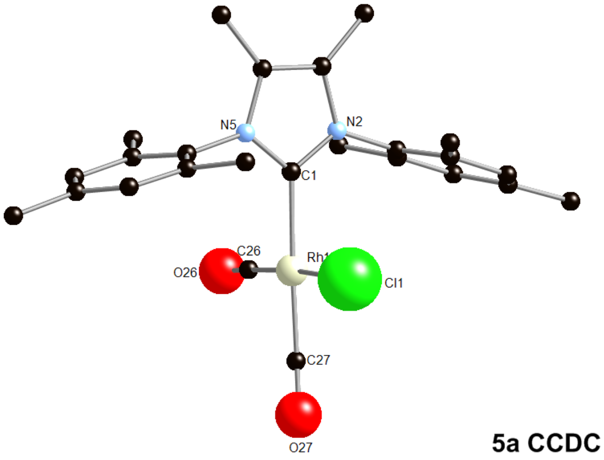

5a CCDC 793640

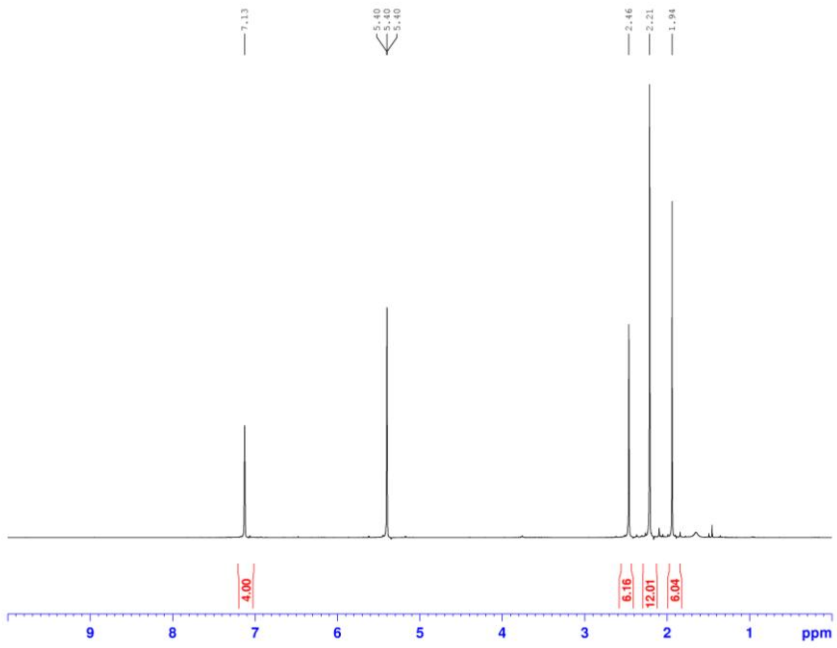
7.13
5.40
5.40
5.40
2.46
2.21
1.94
4.00
6.16
12.01
6.04
9
8
7
6
5
4
3
2
1
ppm

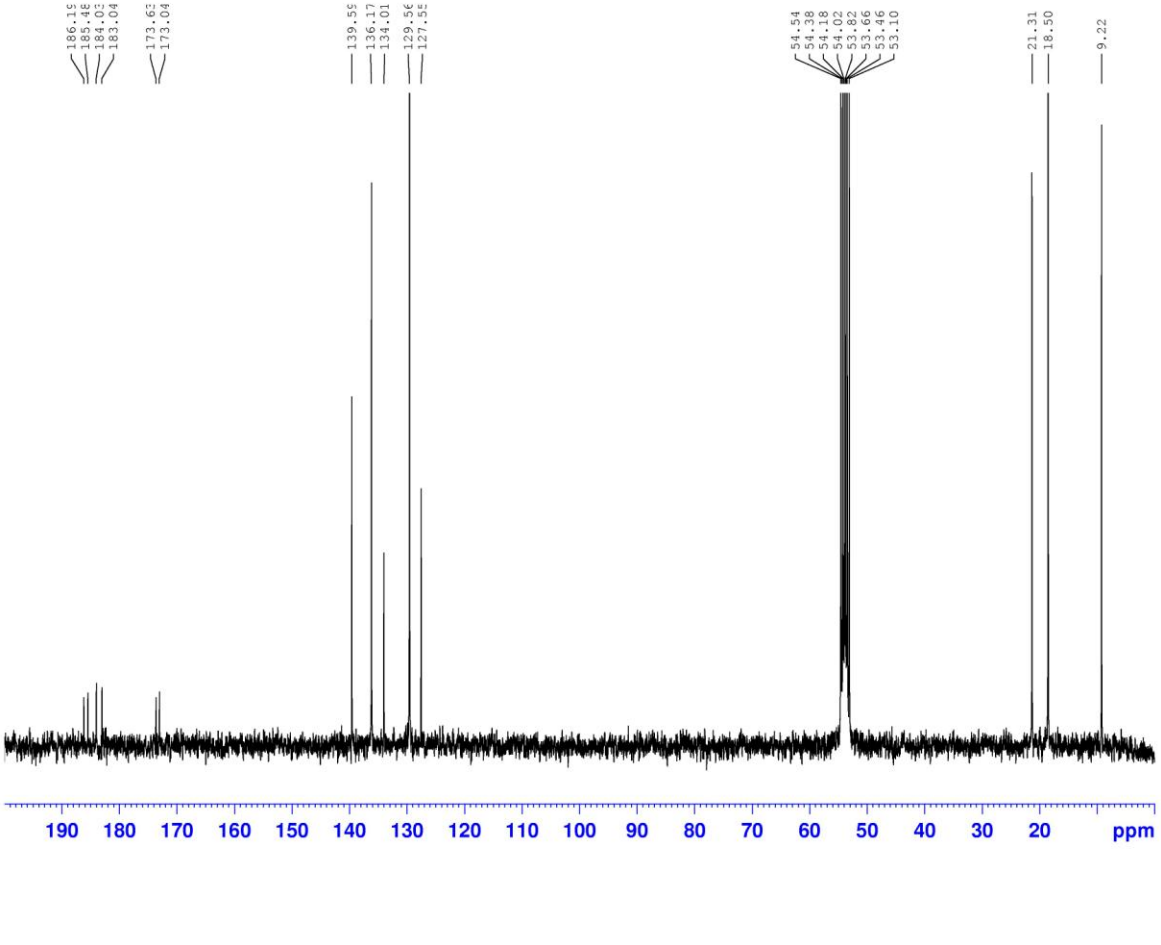
186.19
185.48
184.03
183.04
173.63
173.04
139.59
136.17
134.01
129.56
127.55
54.54
54.38
54.18
54.02
53.82
53.66
53.46
53.10
21.31
18.50
9.22
190
180
170
160
150
140
130
120
110
100
90
80
70
60
50
40
30
20
ppm

Synthesis of [RhCl(CO)$_2$(IMes)] (5b)

In the glovebox, in a vial with a solution of [Rh(CO)$_2$Cl]$_2$ (50 mg, 0.127 mmol) in 5mL of THF, a solution of free IMes (78.0 mg, 0.255 mmol) was added dropwise, the reaction mixture was stirred for 4h, take out of the glovebox and the solvents removed under vacuum. The remaining solid was washed with pentane (3 x 10mL) and dried under vacuum to afford **4b** as a pale yellow solid. Suitable crystals for single X-ray diffraction were grown by vapour diffusion of pentane into a concentrated solution of **4b** in THF (101.8 mg, 0.203 mmol, 80%). ^{1}H NMR (CD$_2$Cl$_2$, 400 MHz): δ = 7.17 (s, 2H, H^{Ar}), 7.07 (s, 4H), 2.41 (s, 6H, *p*-CH$_3^{Mes}$), 2.22 ppm (s, 12H, *o*-CH$_3^{Mes}$). ^{13}C NMR (101 MHz, CD$_2$Cl$_2$-*d*$_2$): δ ppm 185.61 (d, *J* = 57.22 Hz, 1 C) 183.12 (d, *J* = 80.70 Hz, 1C) 177.17 (d, *J* = 46.95 Hz, 1C) 139.77 (s, 2C) 135.38–136.06 (m, 6C) 129.49 (s, 4CH) 124.34 (s, 2CH) 21.29 (s, 2CH$_3$) 18.59 (s, 4CH$_3$) IR (ν_{CO}) in CH$_2$Cl$_2$: 2079.13, 1996.02 cm^{-1}. Anal. calcd for C$_{23}$H$_{24}$ClN$_2$O$_2$Rh (MW 498.81): C, 55.38; H, 4.85; N, 5.62. Found: C, 55.65; H, 4.65; N, 5.63.

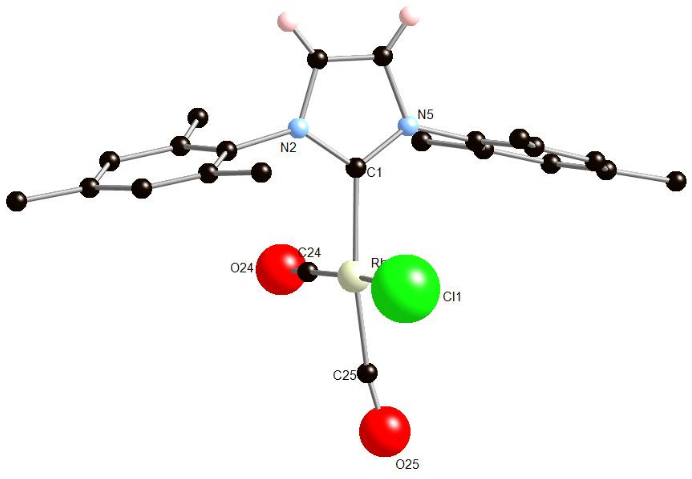

5b CCDC 793641

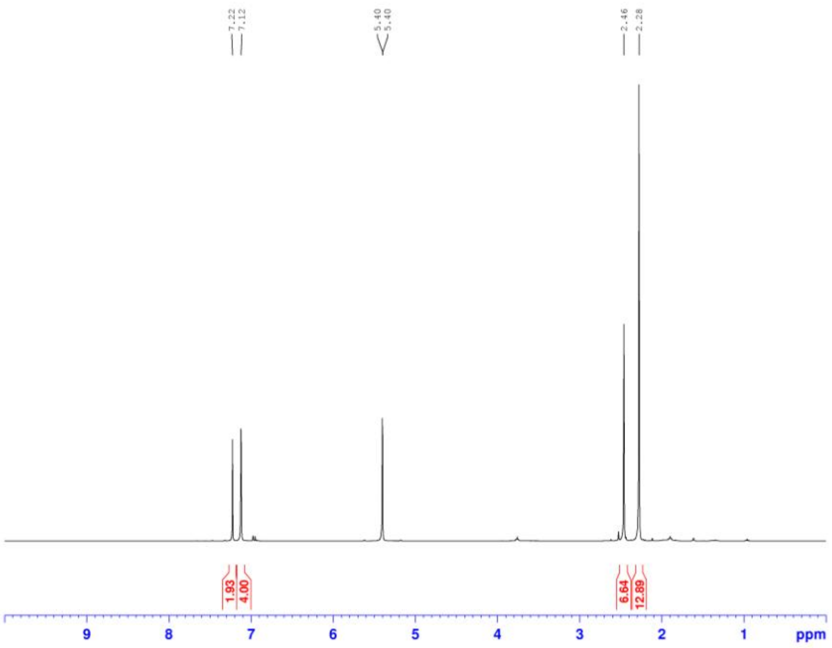
7.22
7.12
5.40
5.40
2.46
2.28
1.93
4.00
6.64
12.89
9 8 7 6 5 4 3 2 1 ppm

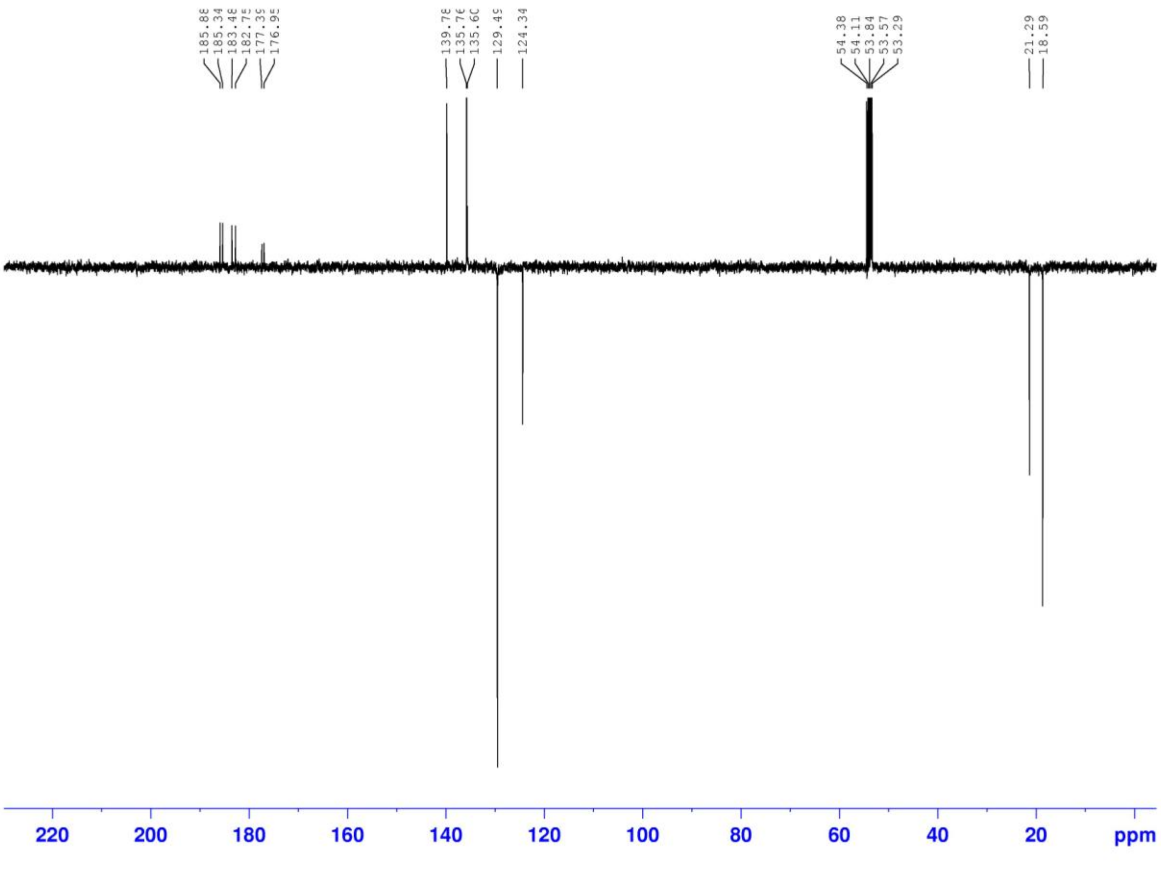
185.88
185.34
183.48
182.75
177.39
176.95
139.78
135.76
135.60
129.49
124.34
54.38
54.11
53.84
53.57
53.29
21.29
18.59
220 200 180 160 140 120 100 80 60 40 20 ppm

Synthesis of $[RhCl(CO)_2(IMesBr)]$ (5c)

In the glovebox, in a vial with free IMes (78.0 mg, 0.255 mmol) and 2 mL of THF, a solution of carbontetrabromide (169.0 mg, 0.510 mmol) in 2 mL of THF was added dropwise and stirred for 2 h. This mixture was then added to a solution of $[Rh(CO)_2Cl]_2$ (50 mg, 0.127 mmol) in 5 mL of THF, and stirred for 4 h, take out of the glovebox and the solvents removed under vacuum. The remaining solid was washed with pentane (3 x 10 mL) and dried under vacuum to afford **4c** as a yellow solid. Suitable crystals for single X-ray diffraction were grown by vapour diffusion of pentane into a concentrated solution of **4c** in DCM (118.4 mg, 0.18 mmol, 71%). ^{1}H NMR (400 MHz, CD_2Cl_2): δ ppm 7.00 (s, 4H, H^{Ar}) 2.32 (s, 6H, *p*-CH_3^{Mes}) 2.09 (s, 12H, *o*-CH_3^{Mes}). ^{13}C NMR (101 MHz, CD_2Cl_2): δ ppm 185.14 (d, J = 58.69 Hz, 1C) 183.01 (d, J = 79.23 Hz, 1C) 180.77 (d, J = 46.22 Hz, 1C) 141.04 (s, 2C) 136.48 (s, 4C) 134.13 (s, 2C) 129. 87 (m, 4CH) 110.59 (s, 2C) 21.55 (s, $2CH_3$) 18.75 (m, $4CH_3$). IR (ν_{CO}) in CH_2Cl_2: 2082.88, 1999.83 cm^{-1}. Anal. calcd for $C_{23}H_{22}Br_2ClN_2O_2Rh$ (MW 656.60): C, 42.07; H, 3.38; N, 4.27. Found: C, 41.62; H, 3.42; N, 4.06.

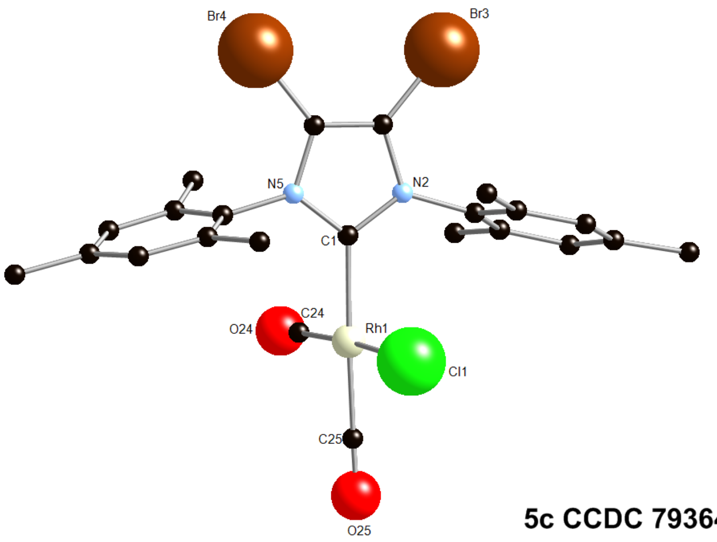

5c CCDC 793642

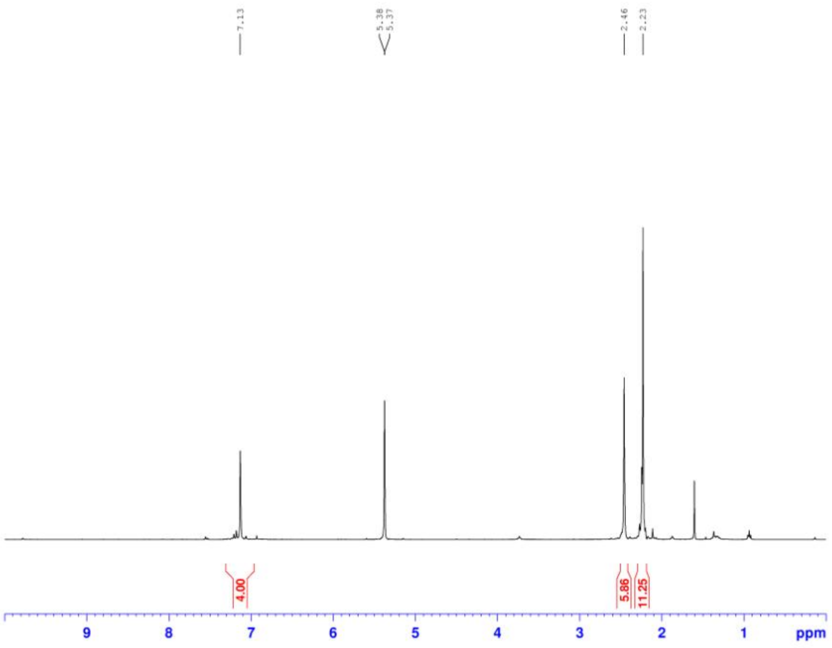
7.13
5.38
5.37
2.46
2.23
4.00
5.86
11.25
9
8
7
6
5
4
3
2
1
ppm

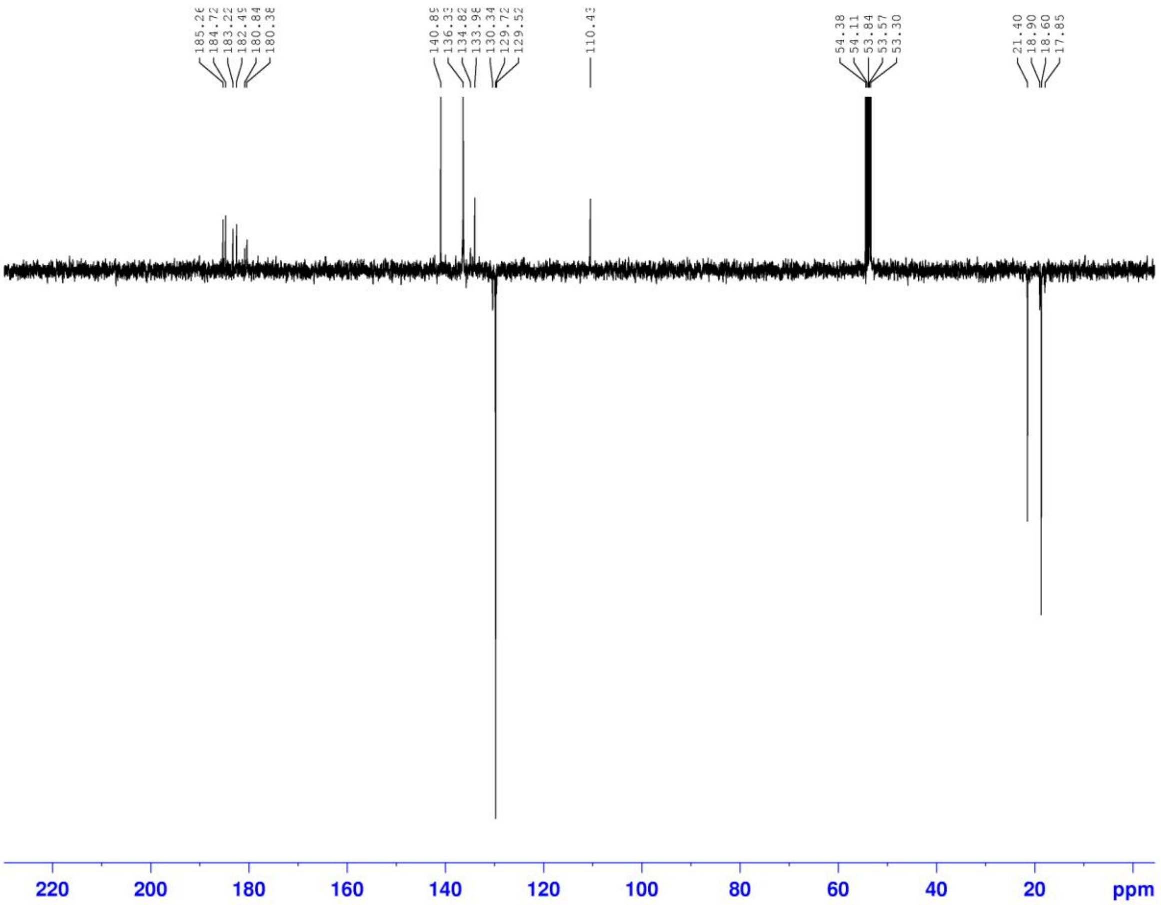
185.26
184.72
183.22
182.49
180.84
180.38
140.85
136.33
134.82
133.98
130.34
129.72
129.52
110.43
54.38
54.11
53.84
53.57
53.30
21.40
18.90
18.60
17.85
220
200
180
160
140
120
100
80
60
40
20
ppm

Synthesis of [RhCl(CO)$_2$(IMesCl)] (5d)

In the glovebox, in a vial with free IMes (78.0 mg, 0.255 mmol) and 2 mL of THF, a solution of carbontetrachloride (78.0 mg, 0.510 mmol) in 2 mL of THF was added dropwise and stirred for 2 h. This mixture was then added to a solution of [Rh(CO)$_2$Cl]$_2$ (50 mg, 0.127 mmol) in 5 mL of THF, and stirred for 4 h, take out of the glovebox and the solvents removed under vacuum. The remaining solid was washed with pentane (3 x 10 mL) and dried under vacuum to afford **4c** as a yellow solid (108.6 mg, 0.19 mmol, 75%). Suitable crystals for single X-ray diffraction were grown by vapour diffusion of pentane into a concentrated solution of **4d** in DCM. ^{1}H NMR (400 MHz, CD_2Cl_2): δ ppm 7.08 (br s, 4H, H^{Ar}) 2.40 (br s., 6H, *p*-CH_3^{Mes}) 2.19 (br s, 12H, *o*-CH_3^{Mes}) ^{13}C NMR (101 MHz, CD_2Cl_2): δ ppm 185.08 (d, J = 55.75 Hz, 1C) 182.78 (d, J = 73.36 Hz, 1C) 178.75 (d, J = 46.22 Hz, 1C) 141.01 (s, 4C) 136.42 (br s, 4C) 132.58 (s, 2C) 129.77 (s, 4CH) 119.55 (br s, 2C) 21.39 (s, $2CH_3$) 18.54 (s, $4CH_3$) IR (ν_{CO}) in CH_2Cl_2: 2084.73, 2000.23 cm^{-1}. Anal. calcd for $C_{23}H_{22}Cl_3N_2O_2Rh$ (MW 567.70): C, 48.66; H, 3.91; N, 4.93. Found: C, 48.32; H, 3. 86; N, 4.28

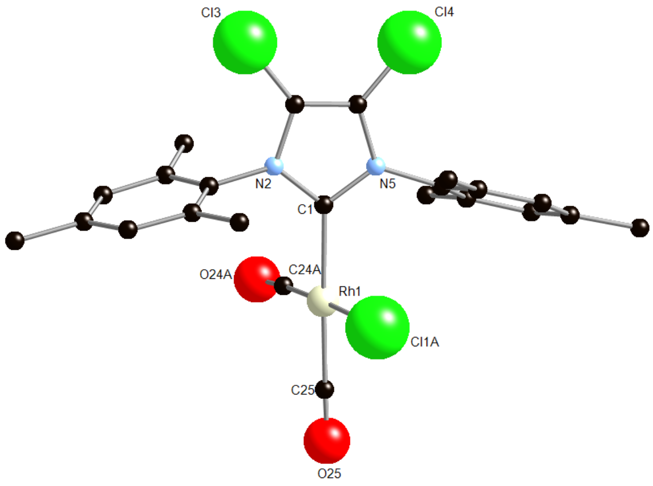

5d CCDC 793643

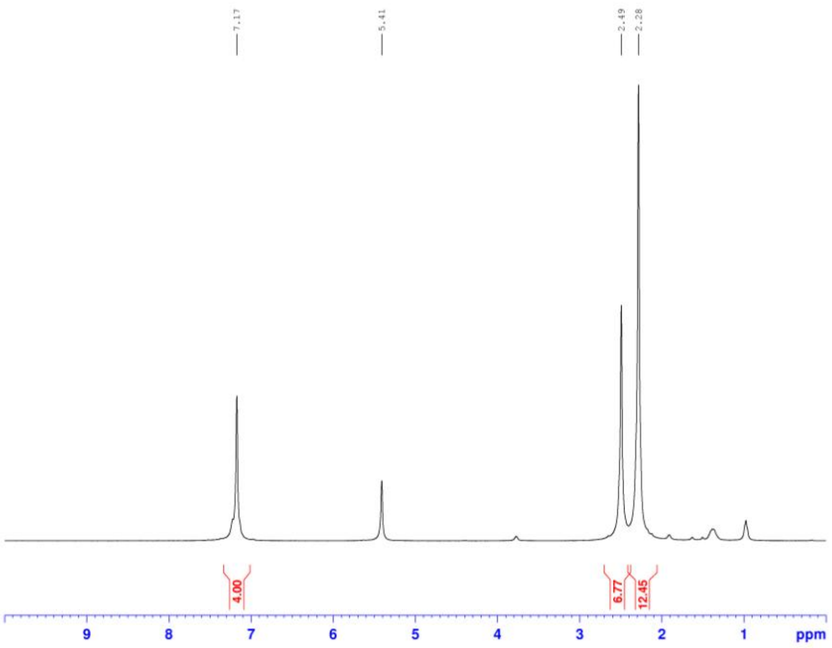
7.17
5.41
2.49
2.28
4.00
6.77
12.45
9 8 7 6 5 4 3 2 1 ppm

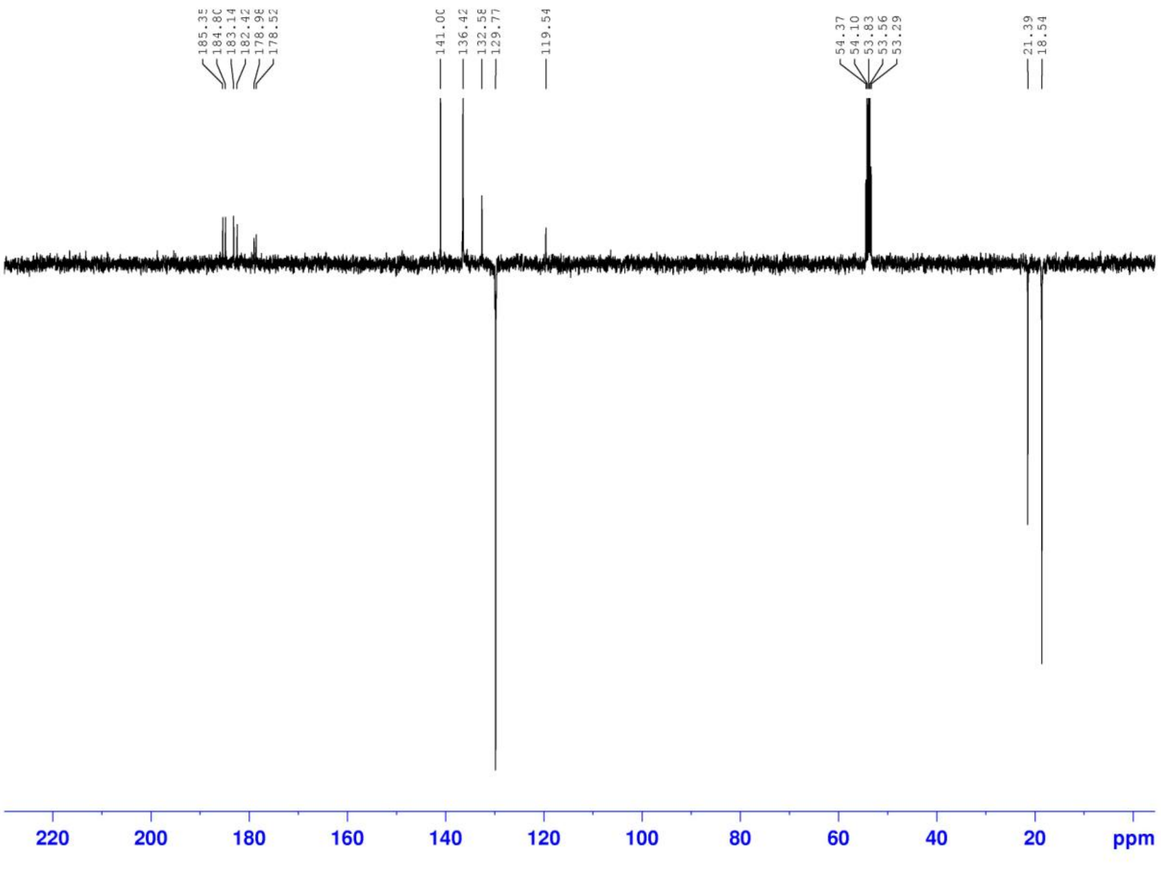
185.35
184.80
183.14
182.42
178.98
178.52
141.00
136.42
132.58
129.77
119.54
54.37
54.10
53.83
53.56
53.29
21.39
18.54
220 200 180 160 140 120 100 80 60 40 20 ppm

Synthesis of [$RuCl_2$(IMesMe)(PCy_3)(Ind)] (6a)

In the glovebox, [$RuCl_2(PCy_3)_2$(Ind)] (461.5 mg, 0.5 mmol) and free IMesMe (166.3 mg, 0.5 mmol) were weighted, then hexane (30 mL) was added, the reaction mixture was heated at 60 ˚C for 3 h outside the glovebox. After solvent evaporation, the remaining solid was purified by column chromatography on silica gel (Hexane:Et_2O, 9:1), affording **5a** as a red solid (253.1 mg, 0.26 mmol, 52%).

^{1}H NMR (CD_2Cl_2, 400 MHz): δ ppm 8.48 (d, J = 7.34 Hz, 1H) 7.62–7.68 (m, 2 H) 7.43 (d, J = 7.52 Hz, 1 H) 7.29–7.37 (m, 2H) 7.14–7.21 (m, 2H) 7.10 (d, J = 7.52 Hz, 1H) 7.01 (s, 2H) 6.39 (s, 1H) 5.94 (s, 1H) 2.30 (d, J = 2.56 Hz, 9H) 1.85 (s, 3H) 1.78 (s, 3H) 1.68 (s, 3H) 1.64 (s, 3H) 1.51 (s, 5H) 1.31–1.48 (m, 13H) 1.15–1.24 (m, 3H) 0.84–1.08 (m, 17H) 0.74–0.83 (m, 5H). ^{31}P NMR (CD_2Cl_2, 121 MHz) δ ppm 27.07 (s). ^{13}C NMR (CD_2Cl_2,101 MHz): δ = 291.4 (d, J = 8.3), 181.5 (d, J = 79.0), 145.3, 141.3, 139.4, 139.1, 138.9, 138.3, 137.8, 137.6, 136.7, 136.5, 136.2, 135.3, 134.2, 129.9, 129.3, 129.1, 128.9, 128.6, 128.3, 128.2, 127.9, 127.7, 127.1, 126.5, 116.2, 33.4, 33.2, 29.8, 29.7, 28.3, 28.2, 28.2, 28.1, 27.0, 26.6, 21.4, 21.2, 20.2, 20.1, 18.8, 18.7, 9.6, 9.0 ppm. Anal. calcd for $C_{56}H_{71}Cl_2N_2PRu$ (MW 975.13): C, 68.98; H, 7.34; N, 2. 87. Found: C, 69.47; H, 7.47; N, 2.68

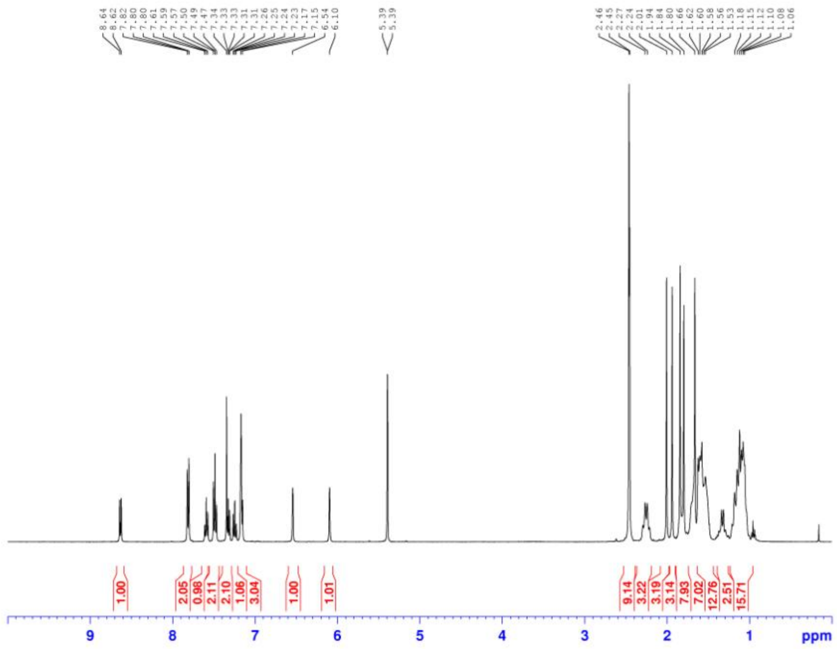

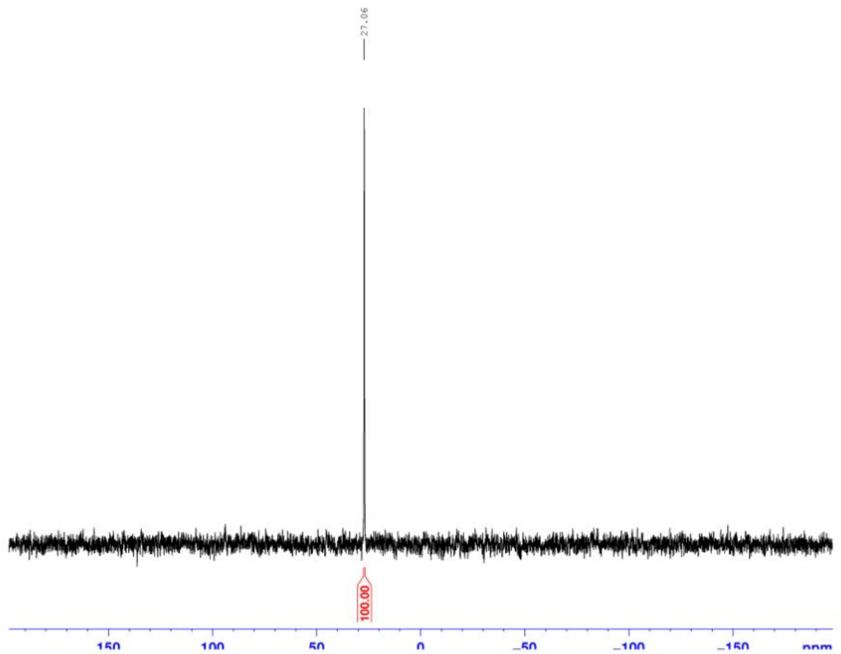

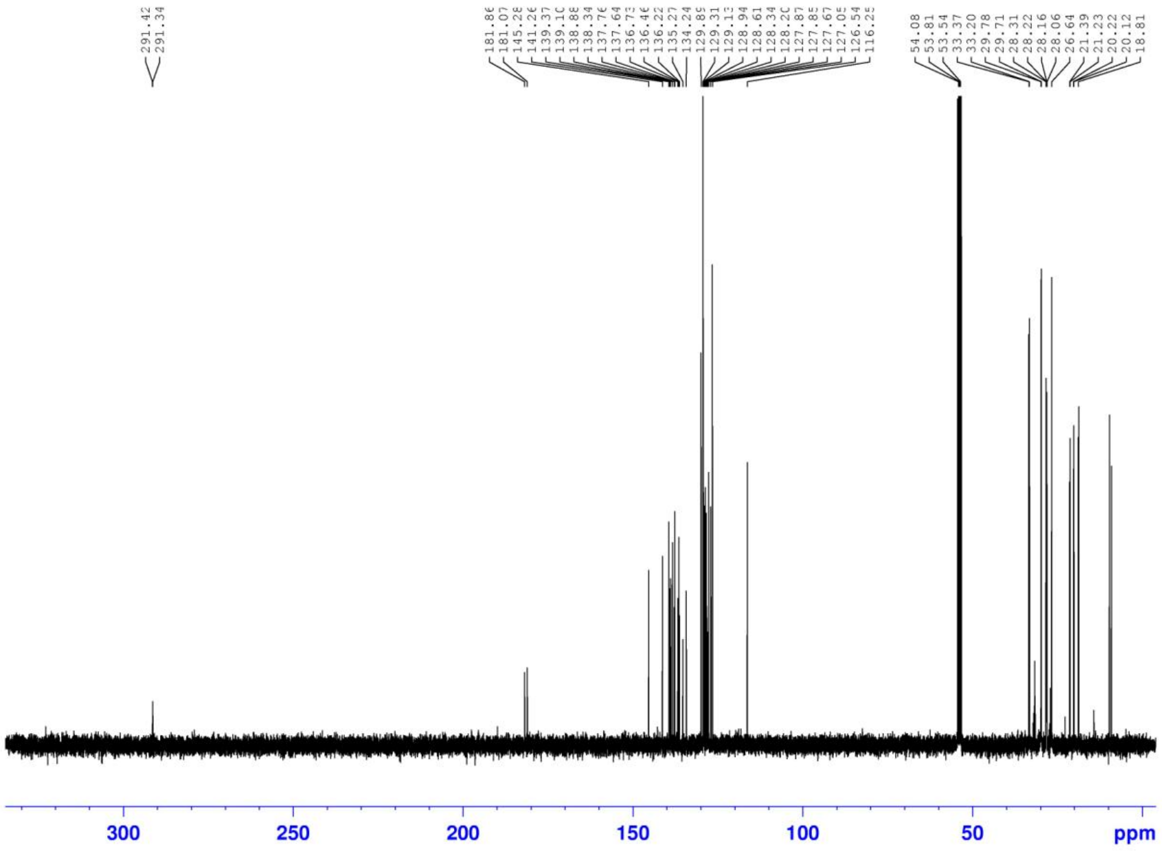

Synthesis of [$RuCl_2$(IMesBr)(PCy_3)(Ind)] (6c)

To a solution of IMesBF_4 (800 mg, 2 mmol) in THF (25 mL), sodium hydride (100 mg, 4 mmol) and potassium *tert*-butoxide (1 spatula) were added, the suspension was stirred overnight, and filtered under argon to remove the excess of NaH. To the filtrate, CBr_4 (1.34 g, 4 mmol) was added, after stirring for 1 h and removal of the solvents to afford a black solid. [$RuCl_2$(PCy_3)$_2$(Ind)] (1.23 g, 1.5 mmol) and hexane (25 mL) were added, the reaction mixture was heated at 70 ˚C for 3 h, filtration through silica gel with DCM as a solvent and recrystallization with Et_2O afforded the **5c** as a dark red solid (900 mg, 0. 81 mmol, 54%).

^{1}H NMR (CD_2Cl_2, 300 MHz): δ = 8.53 (d, J = 7.3 Hz, 1H), 7.65–7.87 (m, 2H), 7.50–7.65 (m, 2H), 7.38–7.50 (m, 3H), 6.93–7.38 (m, 10H), 6.51 (s, 1H), 6.07 (s, 1H), 2.33–2.55 (m, 11H), 2.09–2.26 (m, 4H), 2.00 (s, 3H), 1.88 (s, 3H), 1.82 (s, 3H), 1.36–1.73 (m, 19H), 1.26 (s, 2H), 0.89–1.21 ppm (m, 18H). ^{31}P NMR (CD_2Cl_2, 121 MHz): δ ppm 27.28 (s). ^{13}C NMR (75 MHz, CD_2Cl_2): δ = 279.1, 188.8, 145.1, 140.1, 139.6, 138.4, 138.2, 138.0, 136.0, 135.7, 128.9, 128.3, 127.9, 127.6, 126.9, 126.5, 125.4, 115.3, 109.2, 32.4, 32.2, 28.9, 28.6, 28.6, 27.1, 27.0, 26.9, 25.5, 20.3, 20.2, 19.2, 19.1, 17.7, 17.6 ppm. Anal. calcd for $C_{54}H_{65}Br_2Cl_2N_2PRu$ (MW 1104. 87): C, 58.70; H, 5.93; N, 2. 54. Found: C, 58.50; H, 5.83. 10; N, 2.38.

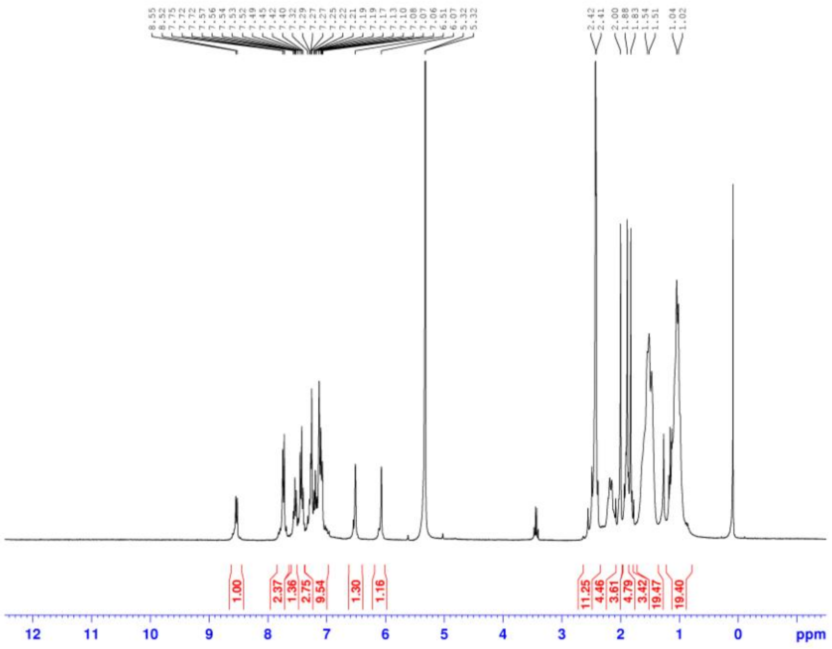

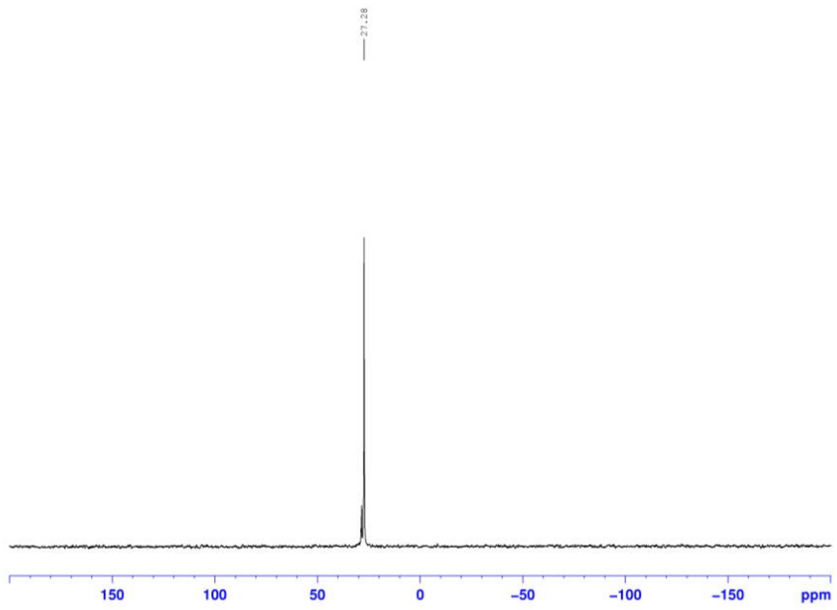

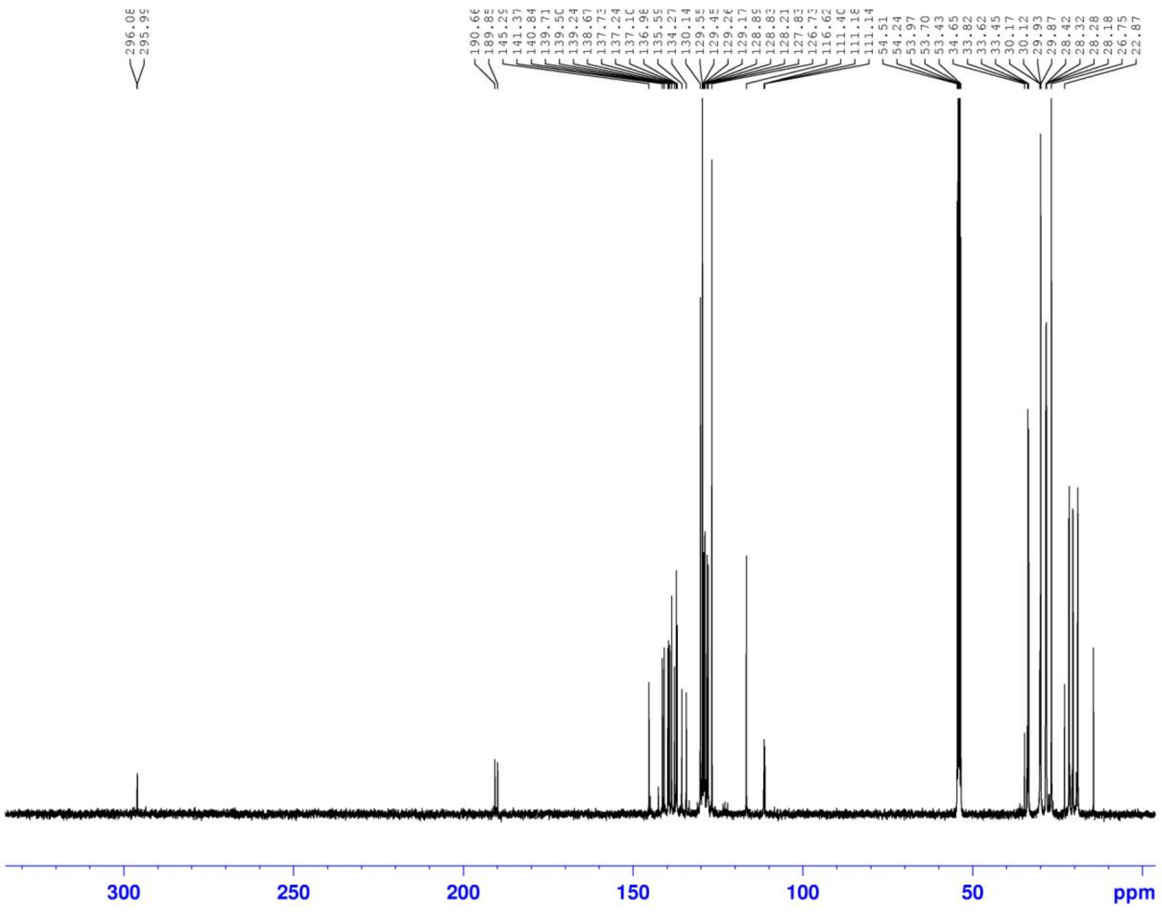

Synthesis of [$RuCl_2$(IMesCl)(PCy_3)(Ind)] (6d)

To a solution of $IMesBF_4$ (3.26 g, 8.3 mmol) in THF (50 mL), sodium hydride (3.984 g, 16.6 mmol), and potassium *tert*-butoxide (1 spatula) were added, the suspension was stirred overnight, and filtered under argon to remove the excess of NaH. To the filtrate, CCl_4 (1.6 mL g, 16.6 mmol) was added, after stirring for 1 h and removal of the solvents to afford a black solid. [$RuCl_2(PCy_3)_2$(Ind)] (1.23 g, 1.5 mmol) and toluene (50 mL) were added, the reaction mixture was heated at 70 ˚C for 3 h, filtration through silica gel with DCM as a solvent and recrystallization with hexane afforded the **5c** as a dark red solid (4.99 g, 4.9 mmol, 79%).

^{1}H NMR (CD_2Cl_2 ,300MHz): δ = 8.52 (dd, J = 7.4, 0.9 Hz, 1H), 7.68–7.79 (m, 3H), 7.49–7.58 (m, 2H), 7.38–7.46 (m, 3H), 7.23–7.30 (m, 3H), 7.16–7.23 (m, 1H), 7.13 (s, 2H), 7.05–7.11 (m, 2H), 6.51 (s, 1H), 6.08 (s, 1H), 2.34–2.48 (m, 12H), 2.08–2.29 (m, 4H), 2.01 (s, 4H), 1.88 (s, 4H), 1.84 (s, 4H), 1.38–1.71 (m, 20H), 0.92–1.21 (m, 20H), 0.76–0.90 ppm (m, 4 H). ^{31}P NMR (CD_2Cl_2, 121 MHz): δ = 26.55 ppm (s). ^{13}C NMR (CD_2Cl_2, 75 MHz): δ = 296.1 (d, J = 9.36 Hz) 188.6 (d, J = 82.19 Hz), 145.3, 141.4, 141.0, 139.8, 139.6, 139.4, 138.7, 137.8, 137.4, 137.3, 137.1, 134.0, 132.8, 130.2, 129.6, 129.5, 129.3, 128.9, 128.3, 127.9, 126.8, 120.3, 119.7, 116.7, 33.7, 33.5, 30.0, 28.4, 28.3, 28.2, 26.8, 21.7, 21.5, 20.3, 18.9, 18.8 ppm. Anal. calcd for $C_{56}H_{65}Cl_4N_2PRu$ (MW 975.13): C, 63.84; H, 6.45; N, 2. 76. Found: C, 63.62; H, 6.45; N, 2.76.

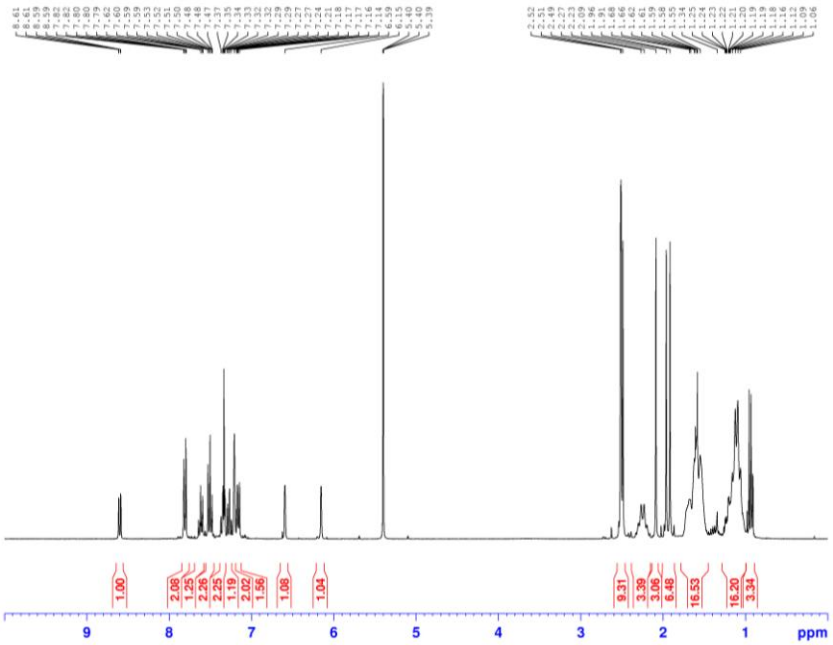

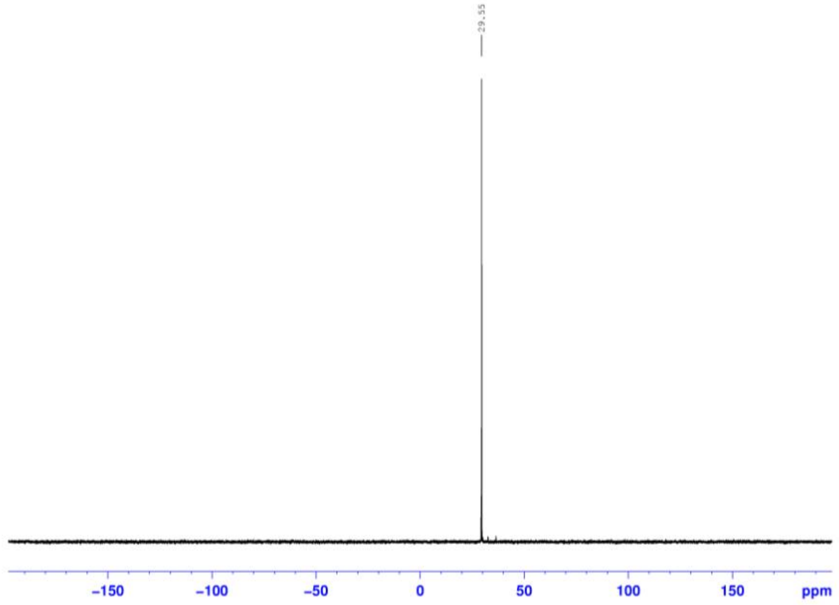
29.55
-150
-100
-50
0
50
100
150
ppm

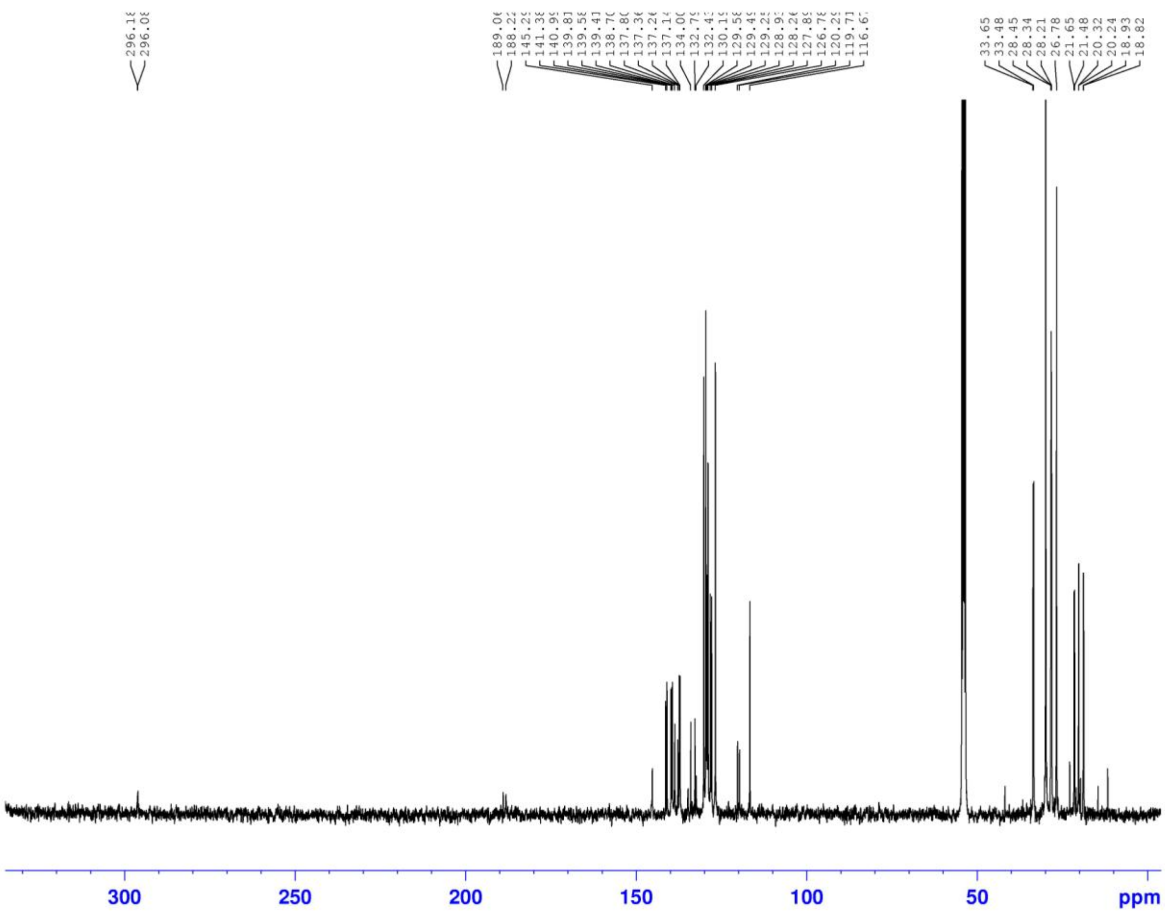
33.65
33.48
28.45
28.34
28.21
26.78
21.65
21.48
20.32
20.24
18.93
18.82
300
250
200
150
100
50
ppm

General procedure for RCM reactions: A Schlenk flask under nitrogen was charged with the substrate (0.5 mmol) and dry dichloromethane (5 mL, c = 0.1 M), then the catalyst (5 x 10^{-3} mmol) was added. The reaction mixture was magnetically stirred at the desired temperature and the progress of the reaction was monitored by TLC. After completion of the reaction, the volatiles were removed under vacuum and the crude reaction mixture was analyzed by ^{1}H NMR spectroscopy. The crude residue was purified by flash column chromatography (pentane:ether 9:1) to yield the pure product.

1. Jafarpour, L.; Schanz, H.-J.; Stevens, E. D.; Nolan, S. P. *Organometallics* **1999**, *18*, 5416–5419.
2. Clavier, H.; Nolan, S. P. *Chem.−Eur. J.* **2007**, *13*, 8029.
3. Chang, S.; Grubbs, R. H. *J. Org. Chem.* **1998**, *63*, 864-866.
4. Bhar, S.; Chaudhuri, S. K.; Sahu, S. G.; Panja, C. *Tetrahedron* **2001**, *57*, 9011.
5. Broggi, J.; Urbina-Blanco, C. A.; Clavier, H.; Leitgeb, A.; Slugovc, C.; Slawin, A. M. Z.; Nolan, S. P. *Chem. Eur. J.* **2010**, *16*, 9215–9225.
6. Fürstner, A.; Ackermann, L.; Gabor, B.; Goddard, R.; Lehmann, C. W.; Mynott, R.; Stelzer, F.; Thiel, O. R. *Chem. Eur. J.* **2001**, *7*, 3236–3253.
